# Case Report: Mepolizumab in the treatment of idiopathic chronic eosinophilic pneumonia

**DOI:** 10.12688/f1000research.130939.3

**Published:** 2023-09-05

**Authors:** Selsabil Daboussi, Samia Essebaa, Samira Mhamdi, Chiraz Aichaouia, Ghedira Hela, Aida Ayadi, Moetemri Zied

**Affiliations:** 1 University of Tunis El Manar, Tunis, Tunisia; 2 Pneumology, Military Hospital of Tunis, Tunis, 1008, Tunisia; 3 Hematology, Military Hospital of Tunis, Tunis, 1008, Tunisia; 4 Pathology, Abderrahmen Mami Hospital,, Ariana, 2080, Tunisia

**Keywords:** Mepolizumab, Idiopathic chronic eosinophilic pneumonia, Corticosteroid, Treatment

## Abstract

Idiopathic chronic eosinophilic pneumonia (ICEP) is a rare interstitial lung disease of unknown cause. It usually responds well to systemic corticosteroid therapy, but relapses are frequent. We describe two cases of 21- and 27-year-old patients, presenting with dyspnea. The diagnosis of steroid-relapsing and steroid-dependent ICEP was made respectively. Mepolizumab was prescribed to both patients. This treatment resulted in successful long-term disease management with much fewer side effects than a traditional corticosteroid therapy.

## Introduction

Idiopathic chronic eosinophilic pneumonia (ICEP) is a rare interstitial lung disease of unknown origin, associated with abnormal eosinophilic lung infiltration. ^1^^–^^3^ Clinical presentation is non-specific. It is characterized by chronic respiratory symptoms, peripheral infiltrates, blood and/or alveolar eosinophilia, and exclusion of other eosinophilic disorders. ^2^^–^^5^ Systemic corticosteroids are the first line recommended treatment. They are efficient, but relapses are frequent. Relapses may occur when the treatment is stopped or when the dose is reduced. ^2^

Mepolizumab is a humanized monoclonal antibody that specifically targets interleukin 5 (IL-5) and neutralizes its effect. Two cases of successfully treated ICEP with mepolizumab are presented. The study was approved by the Ethics Committee Of The Military Hospital In Tunis. Written informed consent was collected from the patients. Anonymity was respected during data treatment.

## Case report 1

**Figure 1.**
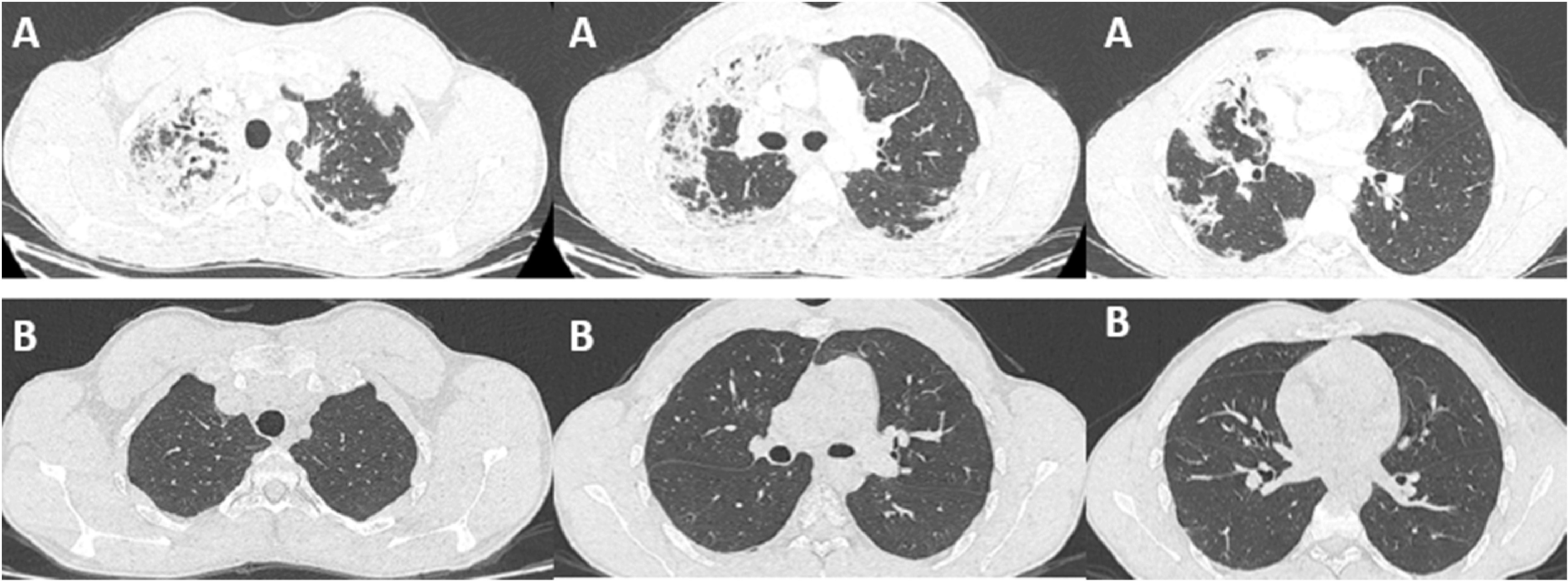
(A) CT chest of the first patient revealing bilateral and peripheral pulmonary infiltrates at the time of diagnosis. (B) CT chest of the first patient after 12 months of mepolizumab and tapering of corticosteroid dose, showing complete resolution of the previous infiltrates.

**Figure 2.**
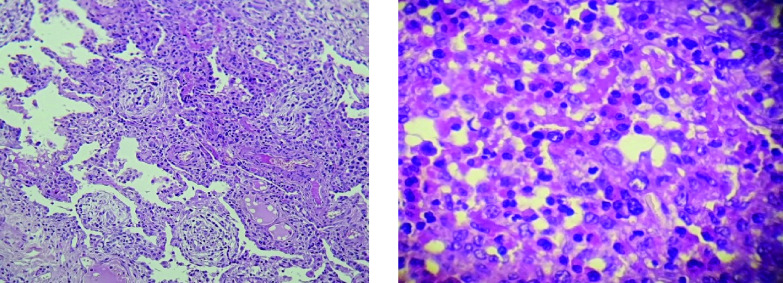
Video-assisted thoracoscopic lung biopsy showing pulmonary eosinophilia with appearances of organizing pneumonia.

**Figure 3.**
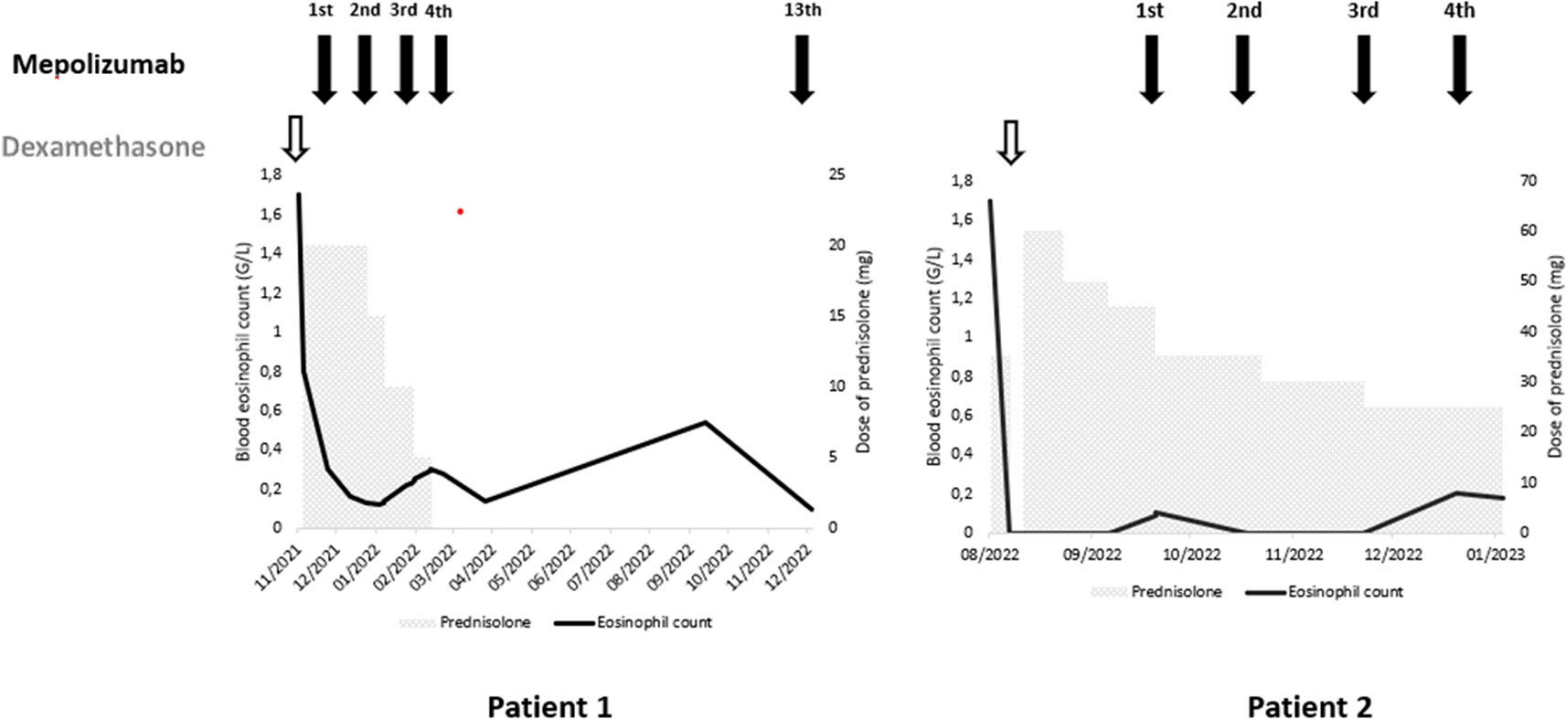
Treatment and evolution of the patients’ eosinophil count.

A 21-year-old patient, high-level athlete, was admitted for dry cough and progressively worsening dyspnea for a month. He also reported 10 kg weight loss during this period. He did not have high fever or wheezing. He was a never smoker and he had no previous medical history. Laboratory findings showed an elevated leukocyte count with a high level of eosinophilia (4.57 G/L, 34.7%). Liver and kidney function tests were normal. Chest computed tomography (CT) showed bilateral and peripheral infiltrative shadows, essentially on the right side ( Figure 1). Bronchoalveolar lavage fluid (BALF) showed an elevated eosinophil percentage (26%). Antineutrophil cytoplasmic antibodies (ANCA) were negative. FIP1L1-PDGFRA and JAK2 V617F mutations were absent. Video-assisted thoracoscopic lung biopsy showed pulmonary eosinophilia with appearances of organizing pneumonia ( Figure 2). The diagnosis of ICEP was established. The patient was managed with high-dose of corticosteroids (20 mg dexamethasone for three days, then 1 mg/kg/day of prednisolone which was tapered over six months). Clinical, biological and radiological improvements were noted. However, the patient relapsed three days after the treatment was stopped. He responded again to steroids. But because of the side effects he experienced from chronic steroid use (muscle wasting, tendon rupture and depression), the decision to initiate an off-label anti-IL5 treatment was made. A monthly 100 mg of subcutaneous mepolizumab was started and oral corticosteroids were gradually stopped. With 12 months of hindsight, he was asymptomatic, the eosinophil counts dropped to normal range ( Figure 3) with a complete radiological clearance. No adverse reaction to the therapy was noted.

## Case report 2

Our second case is a-27-year-old man, active military, admitted for increasing dyspnea, cough and wheezing for a few weeks. He had been diagnosed with asthma one year earlier. He was an ex-smoker of two-five packs per year. Tests showed blood eosinophilia (2G/L, 15%). The C-reactive protein level was 10 mg/L. Chest CT revealed peripheral and diffuse ground glass opacities. The serum Immunoglobulin E level was 502 IU/mL. ANCA were negative. The BALF showed a marked percentage of eosinophils of 31%. Further investigations were unrevealing for parasitic or fungus infections, and hematologic disorders. Hence, the diagnosis of ICEP was made. Systemic corticosteroids were prescribed (4 days of 20 mg of dexamethasone, then 1 mg/kg/day of prednisone, which was tapered over 3 months). His asthma was treated with daily inhaled budesonide and formoterol. The patient responded well to the therapy: a rapid resolution of symptoms was noted, the eosinophils blood count dropped to normal range, and the pulmonary infiltrates completely disappeared. However, attempts at tapering the corticosteroids below 35 mg were met with three relapses in 16 months, which were associated with clinical worsening, reascension of eosinophil count, recurrence of pulmonary lesions and multiple hospital admissions. A high glucose level due to the chronic steroid use was noted. Subcutaneous mepolizumab 100 mg monthly was initiated. A gradual decrease of oral steroids was well tolerated ( Figure 3). The patient is currently at four months of overlap of anti-IL5 and tapering doses of corticosteroids. With a current dose of 20 mg of prednisone, no relapse has occurred.

## Discussion

Eosinophils play a major role in the pathogenesis of ICEP. The IL-5 is a cytokine involved in the production, maturation and release of eosinophils from the bone marrow. Therefore, using a therapy that specifically targets eosinophil activity and proliferation could help treating ICEP. ^1^^,^^3^

Many reports support the use of anti-IL5/5R agents - such as mepolizumab, reslizumab or benralizumab – for the treatment of relapsing and/or corticosteroid dependent/intolerant ICEP. ^2^^,^^4^^–^^7^ These agents have been clinically proven to be effective for eosinophilic asthma, eosinophilic granulomatosis with polyangiitis (EGPA), and FIP1L1-PDGFRA-negative hypereosinophilic syndrome (HES). ^4^

Targeting IL-5 cytokin in patients with ICEP was firstly done by To and al. in 2018. The suppression of local levels of IL-5 and infiltration of eosinophils with mepolizumab resulted in the resolution of CT findings and decreased symptoms. ^5^ In a recent study conducted by Delcros *et al.* - which is the largest cohort of ICEP patients treated with anti-IL5/5R-, no relapses were reported with a median follow up of 13 months. In that study, median annual rate of severe asthma exacerbations decreased from 0.15 to 0, median blood eosinophil count dropped to normal range and a complete disappearance of pulmonary infiltrates was noted in 71% of patients. ^4^

For our first patient, the diagnosis of ICEP was made based on compatible radio-clinical and histopathological presentation. But for our second patient who had asthma, the possibility of EGPA was discussed even in the absence of systemic manifestations and of ANCA. Although a few cases of histologically proven ANCA-negative lung-limited EGPA were reported, we did not approve the use of lung biopsy for our patient due to the rarity of this entity and the invasive nature of the procedure. ^8^

In our patients, the search for alternative therapies was due to an attempt to minimize corticosteroid use. Mepolizumab was chosen due to its availability and the body of evidence to support its use. We prescribed mepolizumab at a monthly dose of 100 mg subcutaneously, which is the dosage used to treat eosinophilic asthma. This dose is three times lower than that used for EGPA and HES. ^2^^,^^9^ Both our patients had a rapid decrease of the blood eosinophil count along a significant clinical and radiologic improvement. Corticosteroid tapering without relapse was also allowed. In the study of Brenard *et al.*, the median daily corticosteroid dose dropped from 5 mg prednisone (range 0–10) at baseline to 0 (range 0–5) after three months of mepolizumab use. ^7^

The overall safety profile of long-term use of mepolizumab has been reported by numerous studies. ^4^^,^^7^^,^^10^ This medication has far fewer toxicities than systemic corticosteroids and can possibly be used in the long term to prevent relapses of ICEP.

## Conclusion

Mepolizumab and other anti-IL5/5R agents seems to be a both safe and effective option as a primary steroid-sparing therapy for corticosteroid dependent or relapsing ICEP. Further investigations and clinical trials are necessary to establish recommendations and clear protocols.

## Consent for publication

Written informed consent for publication of their clinical details and/or clinical images was obtained from the patients.

## Data Availability

All data underlying the results are available as part of the article and no additional source data are required. Figshare. CARE flow diagram and CARE checklist. DOI: https://doi.org/10.6084/m9.figshare.22236772.v1 Data are available under the terms of the Creative Commons Zero “No rights reserved” data waiver (CC BY 4.0 Public domain dedication).
