# Supplementary material for: Case Report: Mepolizumab in the treatment of idiopathic chronic eosinophilic pneumonia
Source: F1000Res. 2023 Sep 5;12:337. Originally published 2023 Mar 27. [Version 3] doi: 10.12688/f1000research.130939.3 (PMC10483178; doi:10.12688/f1000research.130939.3)
Supplement: Peer review history

# Peer review: Reviewer response for version 3

**DOI:** 10.5256/f1000research.155328.r204136

**Published:** 2023-09-06

**Authors:** Mikio Toyoshima

**Affiliations:** 1 Hamamatsu Rosai Hospital, Hamamatsu, Japan

The authors responded and revised the manuscript adequately.

Are enough details provided of any physical examination and diagnostic tests, treatment given and outcomes?

Partly

Is the case presented with sufficient detail to be useful for other practitioners?

Partly

Is sufficient discussion included of the importance of the findings and their relevance to future understanding of disease processes, diagnosis or treatment?

Yes

Is the background of the case’s history and progression described in sufficient detail?

Partly

Reviewer Expertise:

Interstitial pneumonia, eosinophilic pulmonary diseases, asthma

I confirm that I have read this submission and believe that I have an appropriate level of expertise to confirm that it is of an acceptable scientific standard.

# Peer review: Reviewer response for version 2

**DOI:** 10.5256/f1000research.151431.r199918

**Published:** 2023-08-29

**Authors:** Mikio Toyoshima

**Affiliations:** 1 Hamamatsu Rosai Hospital, Hamamatsu, Japan

General comments

 Daboussi et al. reported  two cases of CEP which was successfully treated with mepolizumab.

 Major comments

 However, there are similar case reports regarding anti-IL-5 and anti-IL-5Rα antibody treatment in CEP.

 Minor comments

1. Onset of CEP is not necessarily chronic, and acute or subacute onset of CEP sometimes seen (Hayakawa H, et al. Chest. 1994;105(5):1462-6.). The description "chronic onset of respiratory symptoms" is inappropriate.
2. The degree of BALF eosinophilia in two patients is mild and biopsy specimen in the first patient dose not show numerous eosinophils. Was lung biopsy performed in the second case? Is diagnosis of CEP in 2 patients correct?

Are enough details provided of any physical examination and diagnostic tests, treatment given and outcomes?

Partly

Is the case presented with sufficient detail to be useful for other practitioners?

Partly

Is sufficient discussion included of the importance of the findings and their relevance to future understanding of disease processes, diagnosis or treatment?

Yes

Is the background of the case’s history and progression described in sufficient detail?

Partly

Reviewer Expertise:

Interstitial pneumonia, eosinophilic pulmonary diseases, asthma

I confirm that I have read this submission and believe that I have an appropriate level of expertise to confirm that it is of an acceptable scientific standard, however I have significant reservations, as outlined above.

## References

[1] : A clinical study of idiopathic eosinophilic pneumonia. *Chest* .1994;105(5) : 10.1378/chest.105.5.1462 1462-6 10.1378/chest.105.5.1462 8181338

# Response

**Published:** 2023-08-29

**Authors:** DABOUSSI SELSABIL

**Affiliations:** Tunis El Manar University, Tunisia

Major comments:

 These cases published remain exceptional and rare cases of a treatment that is proving its efficacy for the treatment of CEP. sharing our positive experience could be benefic.

 In the literature there are about 22 similar cases published.

 Minor comments:

 IAEP differs from ICEP in its acute onset, severity,and the absence of relapse. The presentation is that of acute pneumopathy in an individual with no previous history, with the potential for acute respiratory distress. The first diagnosis is often that of infectious pneumopathy, all the more so when fever and elevated C-reactive protein. Symptoms usually set in within1 week, and always within 1 month.In our two patients, the chronic nature of eosinophilic lung disease is due to symptomatology lasting more than 30 days and to frequent relapses, which meet the definition (ref table 4 Cottin V. Eosinophilic lung. EMC - Pneumologie 2020;0(0):1-11 [Article 6-039-K-10]. )

 The degree of BALF eosinophilia in two patients is respectively 26% and 31% and it respond to the definition of eosinophilic alvéolitis.

 Cordially

# Response

**Published:** 2023-08-29

**Authors:** DABOUSSI SELSABIL

**Affiliations:** Tunis El Manar University, Tunisia

We thank Reviewer2 for his positive feedback

 Idiopathic chronic eosinophilic pneumonia (ICEP) is a rare disease of unknown origin  characterized by chronic respiratory symptoms, pulmonary alveolar opacities (typically  of peripheral distribution) and blood and/or alveolar eosinophilia. These criteria were well verified in our two patients, enabling us to retain the diagnosis after an exhaustive etiological investigation that ruled out all other etiologies.

 Video-assisted thoracoscopic lung biopsy (middle and lower right lobes) for the first patient was carried out with this in mind and confirmed the diagnosis by showing pulmonary eosinophilia with appearances of organizing pneumonia (fig2), (inflammatory infiltrate icn in eosinophilis) leaving no doubt to the diagnosis.

 Idiopathic chronic eosinophilic pneumonia (ICEP) is a chronic orphan disease that has no real treatment alternatives to systemic corticosteroids. Accordingly, anti-eosinophil biologics, such as benralizumab and mepolizumab, which do not have glucocorticoid toxicity, are of potential utility in this disease.

 This manuscript uses an analysis of two clinical cases, to examine clinical responses in ICEP patients treated off-label with anti-eosinophil biologics. 

1. We replaced the inappropriate phrase "It is traditionally diagnosed based on the chronic onset of respiratory symptoms"  by It is characterized by chronic respiratory symptoms.

We hope to have answered your pertinent comments and thank you for your interest in our article

# Peer review: Reviewer response for version 2

**DOI:** 10.5256/f1000research.151431.r182980

**Published:** 2023-08-21

**Authors:** Sevim Bavbek

**Affiliations:** 1 Division of Immunology and Allergy, Department of Chest Diseases, Ankara Universitesi, Ankara, Ankara, Turkey

I am fine with the new revised version. I approve the article for indexing.

Are enough details provided of any physical examination and diagnostic tests, treatment given and outcomes?

Partly

Is the case presented with sufficient detail to be useful for other practitioners?

Partly

Is sufficient discussion included of the importance of the findings and their relevance to future understanding of disease processes, diagnosis or treatment?

Partly

Is the background of the case’s history and progression described in sufficient detail?

Yes

Reviewer Expertise:

NA

I confirm that I have read this submission and believe that I have an appropriate level of expertise to confirm that it is of an acceptable scientific standard.

# Peer review: Reviewer response for version 1

**DOI:** 10.5256/f1000research.143737.r168068

**Published:** 2023-04-04

**Authors:** Sevim Bavbek

**Affiliations:** 1 Division of Immunology and Allergy, Department of Chest Diseases, Ankara Universitesi, Ankara, Ankara, Turkey

I would like to thank you for the opportunity to review this article.

 Idiopathic chronic eosinophilic pneumonia (ICEP) is a rare interstitial lung disease of unknown cause and responds well to systemic corticosteroid therapy, although frequent relapses are reported. Here two cases of CEP patients have been reported.

 The paper might have potential to be indexed but the point below needs to be clarified:

 Cases with CEP in the case report, have been well evaluated and presented but for case 2, presence of EGPA may be an alternative diagnosis. In this case, there is not data about ANCA but anyway ANCA may be negative in cases with eosinophilic dominant without presence of vasculitis yet. This possibility should be discussed. Mepolizumab has also been approved in EGPA

Are enough details provided of any physical examination and diagnostic tests, treatment given and outcomes?

Partly

Is the case presented with sufficient detail to be useful for other practitioners?

Partly

Is sufficient discussion included of the importance of the findings and their relevance to future understanding of disease processes, diagnosis or treatment?

Partly

Is the background of the case’s history and progression described in sufficient detail?

Yes

Reviewer Expertise:

Asthma, severe asthma, biologicals, drug allergy, desensitization to drugs

I confirm that I have read this submission and believe that I have an appropriate level of expertise to confirm that it is of an acceptable scientific standard, however I have significant reservations, as outlined above.

# Response

**Published:** 2023-05-16

**Authors:** DABOUSSI SELSABIL

**Affiliations:** Tunis El Manar University, Tunisia

We would like to thank you for reviewing this article and for providing pertinent comments. For the case 2, the ANCA were negative.

 Indeed, ANCA-negative EGPA is an alternative diagnosis for this patient.

 But he did not present with paranasal abnormalities, cutaneous, neurological, gastrointestinal or cardiovascular symptoms, and did not meet the ACR criteria for the disease.

 Therefore, we retained the diagnosis of ICEP.

 This possibility will be discussed in the revised version of the article.
